# Left lateral intercostal region versus subxiphoid position for pleural drain during elective coronary artery bypass graft surgery: randomized clinical trial

**DOI:** 10.1590/1516-3180.2018.040940119

**Published:** 2019-05-08

**Authors:** Sandra Simon, Christian Coronel, Adriana Silveira de Almeida, Aline Marcadenti

**Affiliations:** I MSc. Registered Nurse, Graduate Program in Health Sciences (Cardiology), Instituto de Cardiologia, Fundação Universitária de Cardiologia (IC/FUC), Porto Alegre (RS), and Registered Nurse, Cardiology and Cardiac Surgery Services, Hospital Nossa Senhora da Conceição Hospital (HNSC), Porto Alegre (RS), Brazil.; II MSc. Physiotherapist, Physical Therapy Service, Surgery Service, Instituto de Cardiologia, Fundação Universitária de Cardiologia (IC/FUC), Porto Alegre (RS), and Professor, La Salle University (Unilasalle Canoas), Canoas (RS), Brazil.; III PhD. Physician and Cardiovascular Surgeon, Cardiology and Cardiac Surgery Services, Hospital Nossa Senhora da Conceição Hospital (HNSC), Porto Alegre (RS), Brazil.; IV PhD. Professor, Graduate Program in Health Sciences (Cardiology), Instituto de Cardiologia, Fundação Universitária de Cardiologia (IC/FUC), Porto Alegre (RS); Professor, Postgraduate Program on Nutrition Sciences, Universidade Federal de Ciências da Saúde de Porto Alegre (UFCSPA), Porto Alegre (RS); and Researcher, Instituto de Pesquisa do Hospital do Coração (HCor), São Paulo (SP), Brazil.

**Keywords:** Myocardial revascularization, Extracorporeal circulation, Respiratory function tests, Pleural effusion, Mammary arteries

## Abstract

**BACKGROUND:**

The pleural drain insertion site after coronary artery bypass graft (CABG) surgery may alter lung function, especially respiratory muscle strength. The main objective of this study was to compare the effectiveness and safety of use of the left lateral intercostal region versus the subxiphoid position for pleural drainage during elective CABG surgery using extracorporeal circulation (ECC).

**DESIGN AND SETTING:**

Randomized trial conducted in a tertiary-level hospital in Porto Alegre, Brazil.

**METHODS:**

48 patients were assigned to group 1 (pleural drain in the left lateral intercostal region) or group 2 (pleural drain in the subxiphoid position). Respiratory muscle strength was measured in terms of maximal inspiratory pressure (MIP) and maximal expiratory pressure (MEP), in cmH_2_O, by means of manovacuometry preoperatively, 24 and 72 hours after drain removal and before discharge from hospital. Painand dyspnea scales, presence of infections, pleural effusion and atelectasis, duration of drain use, drainage volumes and surgical reinterventions were also evaluated.

**RESULTS:**

After adjustments, there were no significant differences between the groups at the end of the study (before discharge), in predicted percentages either for MIP (delta group 1: -17.21% versus delta group 2: -22.26%; P = 0.09) or for MEP (delta group 1: -9.38% versus delta group 2: -13.13%; P = 0.17). Therewere no differences between the groups in relation to other outcomes.

**CONCLUSION:**

There was no difference in maximal respiratory pressures in relation to the pleural drain insertion site among patients who underwent CABG surgery using ECC.

**TRIAL REGISTRATION:**

ReBEc V1111.1159.4447.

## INTRODUCTION

Coronary artery bypass graft (CABG) surgery is associated with higher survival rates and better quality of life among patients with coronary artery disease.^1^^,^^2^ Use of left internal thoracic artery (LITA) grafts has been correlated with long-term benefits,^3^ but this often requires pleurotomy and insertion of tubes to drain the cavity.^4^^,^^5^

Pleural drains can be inserted into the subxiphoid region or the intercostal space with the main objective of maintaining or restoring the negative pressure of the pleural space.^6^ However,they may impair the integrity of the ventilatory system, thereby compromising the respiratory mechanics and gas exchange after surgery.^7^^,^^8^^,^^9^

Respiratory muscle strength may be evaluated through maximal inspiratory pressure (MIP) and maximal expiratory pressure (MEP), which indicate the strength of the inspiratory and expiratory muscle groups respectively.^10^Predictions for MIP and MEP according to age and sex should preferably be considered within their clinical setting, because they may lead to a prognosis of postoperative pulmonary complications like respiratory muscle fatigue or failure.^11^^,^^12^^,^^13^ MIP and MEP can be measured with the aid of a manometer or manovacuometer. In addition to being practical and non-invasive, this equipment has low cost, is easy to apply at the bedside and only requires simple inspiration and expiration movements from the patient.

Studies on individuals undergoing CABG surgery have shown that insertion of the pleural drain in the subxiphoid position can minimize the chance of trauma to the thoracic wall, may preserve respiratory function in the immediate postoperative period and may lead to lower levels of subjective pain, compared with lateral intercostal insertion.^14^^,^^15^^,^^16^^,^^17^^,^^18^^,^^19^^,^^20^ However, most of these studies were conducted among patients undergoing CABG surgery without extracorporeal circulation (ECC),^15^^,^^16^^,^^17^^,^^20^^,^^21^ without prior pulmonary disease,^14^^,^^15^^,^^16^^,^^18^^,^^19^ and with use of spirometry rather than manovacuometry to evaluate respiratory muscle strength.^14^^,^^15^^,^^18^^,^^19^^,^^20^

## OBJECTIVE

The main objective of this study was to compare the effects of pleural drain insertion in the subxiphoid region with insertion in the left lateral intercostal region, on MIP and MEP measured via manovacuometry, among patients undergoing elective CABG surgery with ECC and use of LITA grafts. The secondary aims were to compare pain, dyspnea, infections, pleural effusion, atelectasis, drainage volumes, surgical reintervention and number of hours with the drain between the groups.

## METHODS

### Study design and setting

This was a parallel randomized clinical trial conducted among candidates for CABG with ECC and use of LITA grafts who were admitted to the Cardiology and Cardiac Surgery Service of the Nossa Senhora da Conceição Hospital (HNSC, Porto Alegre, Brazil). The research project was approved by the Research Ethics Committee (REC) of the University Foundation of Cardiology (UP protocol 4904/13) in August 2013 and by the REC of the Conceição Hospital Group under number 14-226 in May 2014. The protocol was registered in the Brazilian Registry of Clinical Trials (ReBEc) under the number V1111.1159.4447. All participants signed an informed consent form. Data collection was carried out from July 2014 to August 2015. We used the CONSORT Statement for reporting this trial.

### Study population

Patients aged between 40 and 80 years, with an indication for CABG surgery with LITA graft^2^ and associated pleurotomy, were included in the study. Patients with severe neuropsychiatric deficits who required concomitant surgical interventions such as valve replacement or aortic surgery, or who presented symptomatic abdominal hernias, stroke prior to or during the study period or any other conditions that impeded use of a manovacuometer (strength deficit, sensory deficit, facial paralysis or pleurocutaneous or pulmonary fistulas), were excluded.

### Randomization and allocation concealment

The block randomization sequence was generated with the aid of the website www.randomization.org and the numbers were allocated through using individual opaque sealed envelopes. Afterchecking the eligibility criteria and after patients had signed the consent form, the participants were allocated to either of two groups (1:1 randomization): group 1: with use of a left lateral drain, inserted at the intersection of the 6^th^ or 7^th^ left intercostal space with the middle axillary line; or group 2: with use of a drain inserted in the subxiphoid region. Only one researcher had access to the randomization list and he did not participate in the enrollment. The patients, surgeons and researchers involved in both allocation and data collection (including the operators who performed the manovacuometry) were aware of the group to which the participants were randomized because these patients were identified by means of a green or orange patch on their hospital bracelet, to indicate to the surgeon which pleural drain insertion site should be used (green label: lateral insertion; orange label: subxiphoid insertion). The clinical cardiologists, radiologists and other professionals who evaluated the patients’ examinations, along with the professionals involved in the statistical analysis, were blinded to the randomization groups.

### Primary outcomes

The primary outcomes were MIP and MEP, in cmH_2_0, evaluated by means of manovacuometry. This was performed in accordance with standardized protocols, at the baseline and at another three times: 24 hours and 72 hours after drain removal and at hospital discharge.^21^^,^^22^ We used an analogue manovacuometer (M120; Globalmed, Porto Alegre, RS, Brazil) that had been certified by the Brazilian standards agency Inmetro and which was capable of making measurements over the range from -150 to +150 cmH_2_0.

Predicted MIP and MEP values were calculated using the equations proposed by Neder et al.^23^ for individuals aged 20 to 80 years, separately for males (MIP = 155.3 - 0.80*height; MEP = 165.4 - 0.81*height) and for females (MIP = 110.4 - 0.49*height; MEP = 115.6 - 0.61*height). The predicted percentages for maximal respiratory pressures were calculated individually based on the formula: (measured MIP or MEP/predicted MIP or MEP)*100.

### Secondary outcomes

#### Subjective degree of pain

The subjective degree of pain at the site of drain insertion was ascertained at 24 hours and 72 hours in the postoperative period and before hospital discharge, with the aid of a visual analogue scale graded from zero to ten,^24^ on which zero represented absence of pain and ten, the most intense pain.

#### Subjective degree of dyspnea

The subjective degree of dyspnea was assessed at 24 hours and 72hours in the postoperative period and before hospital discharge using the modified Borg dyspnea scale,^25^ graded from zero to ten, in which zero characterized absence of dyspnea and ten, the worst sensation of dyspnea.

#### Respiratory and surgical wound infection

Respiratory infection was defined, in accordance with clinical criteria,^26^ as an association of hyperthermia, infectious leukogram and compatible radiological examination. Surgical wound infections were diagnosed from occurrences of local phlogistic signs such as heat, redness, pain, purulent secretion and edema, along with the presence of sternum instability, fever and an infectious leukogram.^26^^,^^27^^,^^28^^,^^29^

#### Pleural effusion and atelectasis

The presence of pleural effusion was evaluated through simple chest x-ray examinations in the posterior-anterior and lateral incidences, which were interpreted by two independent blinded physicians. The first x-ray was performed between 48 hours and 72 hours after drain removal; the second examination was performed between 73 hours and 120 hours after drain removal. Pleural effusion was categorized as absent, small or medium, and was assessed as present on the right or left side. Atelectasis was also evaluated based on radiological examinations and was registered in the medical records.

#### Duration of drain use and pleural drainage

The duration of pleural drain use was recorded as the number of hours. The volumes collected through mediastinal and pleural drainage (in milliliters) were ascertained from the hospital records. The need for medical reinterventions during the hospital stay was evaluated considering the need for pleural drainage after removal of the chest drain, through insertion of a tubular drain or thoracocentesis.

### Other variables

At the first assessment (preoperative), sociodemographic data (sex and age) and clinical data regarding the patients’ current and previous medical histories and use of medications were collected by means of interviews and from hospital records. Existence of a smoking habit was categorized as “current”, “former” (if the individual had stopped smoking for more than a year) or “never”. Alcohol consumption and/or alcohol abuse (consumption of ≥ 30 g/day for men and ≥ 15 g/day for women) was identified from the medical records and clinical history. Patients were deemed to be former alcoholics if they had ceased their abusive consumption of alcoholic beverages more than one year previously. Body mass index (BMI) was calculated as the ratio between body mass (in kilograms) and squared height (in meters) and was expressed as kg/m^2^.

#### Clinical and cardiological history

Clinical and cardiological information were collected from the hospital medical records, including the current disease history(symptoms and their characteristics), previous disease history (hypertension, diabetes mellitus, dyslipidemias, previous myocardial infarction and angina) and previous examinations. Family histories of previous diseases and treatments, such as percutaneous myocardial revascularization or non-cardiological surgery were also taken into consideration.

All the patients underwent a baseline electrocardiogram, which was used for comparative evaluations between the pre and postoperative periods. The professionals involved were blinded to the study objectives. The anesthetic technique used was the same for both groups (balanced general anesthesia, with inhalants and venous agents). Epidural analgesia was not used in any case. Thechest tube caliber was 38 French for both groups.

#### Intraoperative period variables

Intraoperative variables were obtained from the medical records in both the surgical and the intensive care unit, as follows:

- Total duration of ECC: length of time with extracorporeal circulation, in minutes;
- Duration of surgery: length of time, in hours, that elapsed from the arrival of the patient in the surgical room until entry to the intensive care unit (ICU);
- Duration of mechanical ventilation: length of time, in hours, that elapsed between orotracheal intubation of the patient in the surgical room and extubation in the ICU;
- Length of stay in the ICU: length of stay in the ICU, in hours, until referral to the inpatient unit;

Outcomes were collected from the medical records. Myocardial infarction and revascularization followed by death in the same hospital was adjudged to be cardiovascular death.

### Sample calculation

The sample size calculation was performed using the WinPepi software for Windows. Based on data from a previous randomized trial that assessed maximal respiratory pressures among patients undergoing CABG surgery,^16^ we found that we would need a sample size of 20 participants in each group to find a difference in predicted percentage for MIP of 14% between the groups, considering standard deviations of 15% both in group 1 and in group 2, a study power of 80%, and an alpha level of 0.05. After adding 20% to cover for losses, the final sample size would need to be 48 individuals.

### Statistical analyses

The data were recorded in a database in the Excel software, version 2013. The analyses were carried out in the Statistical Package for the Social Sciences (SPSS), version 20 for Windows. All the analyses were performed using the intention-to-treat principle. Continuous variables were described as means and standard deviations (forsymmetrical variables) and/or medians and interquartile ranges (for asymmetrical variables). Categorical variables were described as absolute frequencies and numbers. Student’st test was used to compare means, and the Wilcoxon-Mann-Whitney test to compare medians. Comparisons between proportions were made using Pearson’s chi-square test or Fisher’s exact test. Generalized estimated equation (GEE) tests and Bonferroni post-hoc tests were used for intergroup and intragroup comparisons between different times. A significance level of 5% was used.

## RESULTS

**Figure 1.**
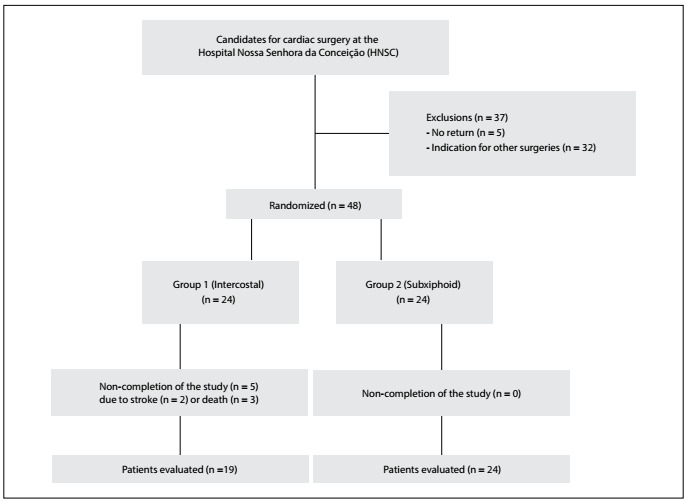
Flowchart of study participants.

During the enrollment period, from July 2014 to August 2015, 85patients were admitted electively or urgently to the Cardiology and Cardiac Surgery Service of the HNSC with a surgical indication and thus were eligible for inclusion in the study. Of these, five were discharged for an elective return and did not return to undergo the CABG procedure, and 32 individuals had indications for other surgeries. In the end, 48 patients underwent randomization (Figure 1). Among these, five patients who were allocated to group 1 did not complete the study: three because of death (twodue to cardiovascular causes, i.e. cardiogenic shock on the first postoperative day, and one due to an infectious cause, consisting of pulmonary sepsis); and two because they suffered incapacitating strokes during the perioperative period. Thus,19patients randomized to the lateral drain group and 24randomized to the subxiphoid drain group completed the study.

Table 1 presents the characteristics of the participants according to the randomized groups at the baseline. There were no significant differences between the groups. The previous surgeries that were identified were valve replacement, prostatectomy, cholecystectomy and mastectomy, with no difference in prevalence between the groups (P = 0.72). The individuals allocated to the lateral group presented higher prevalence of prior myocardial infarction associated with percutaneous revascularization, compared with the subxiphoid group (P = 0.038).

**Table 1.** Baseline characteristics according to study groups [mean ± standard deviation; n (%)]

	Lateral drain N = 24	Subxiphoid drain N = 24	P-value
Male	15 (62.5)	16 (66.7)	0.76*
Age in years	65.58 ± 9.50	61.75 ± 9.58	0.17^‡^
Body mass index in kg/m^2^	27.45 ± 4.72	28.93 ± 3.49	0.22**
Diabetes mellitus	13 (54.2)	10 (41.7)	0.56*
Hypertension	20 (83.3)	23 (95.8)	0.35*
Dyslipidemia	17 (70.8)	21 (87.5)	0.29*
Asthma/chronic obstructive pulmonary disease	4 (16.7)	4 (16.7)	> 0.90*
Chronic kidney disease	2 (8.3)	3 (12.5)	> 0.90*
Peripheral obstructive disease/peripheral arterial obstructive disease	5 (20.5)	2 (8.3)	0.42*
Previous stroke	3 (12.5)	2 (8.3)	> 0.90*
Smoking			0.32*
Current smoker	2 (8.3)	2 (8.3)	
Former smoker	13 (54.2)	8 (33.3)	
Never smoked	9 (37.5)	14 (58.3)	
Alcohol consumption			> 0.90*
Former alcoholism	2 (8.3)	2 (8.3)	
No alcoholism	22 (91.7)	22 (91.7)	
Coronary artery disease			0.038*
Previous acute myocardial infarction	8 (33.3)	3 (12.5)	
Acute myocardial infarction with percutaneous revascularization	3 (12.5)	0	
Angina	9 (37.5)	10 (41.7)	
Angina with percutaneous revascularization	0	4 (16.7)	
No history of coronary artery disease	4 (16.7)	7 (29.2)	
Family history of CAD	3 (12.5)	3 (12.5)	> 0.90**
Reason for hospital admission			> 0.90*
Acute myocardial infarction	9 (37.5)	9 (37.5)	
Angina	15 (62.5)	15 (62.5)	
Lesions identified by means of coronary artery angiography			0.35*
Left coronary trunk	1 (4.2)	0	
Left coronary trunk + other coronary arteries	2 (8.3)	5 (20.8)	
Multiarterial	20 (83.3)	19 (79.1)	
Intra-stent restenosis (previous)	1 (4.2)	0	
Chronic-use medications			
Acetylsalicylic acid	22 (91.7)	19 (79.2)	0.42**
Clopidogrel	7 (29.2)	6 (25)	> 0.90**
Statins	20 (83.3)	17 (70.8)	0.49**
Beta-blockers	16 (66.7)	15 (62.5)	> 0.90**
Resting electrocardiogram			0.36*
Sinus rhythm	22 (91.7)	21 (87.5)	
Atrial fibrillation	1 (4.2)	3 (12.5)	
Others	1 (4.2)	0	

Statistical tests used: *Pearson’s chi-square test; ‡ Student’s t test; **Fisher’s exact test.

Table 2 shows the perioperative data on the patients according to the randomized groups. There were no significant differences in the variables at the preoperative assessment (heart rate, oxygen saturation, maximal respiratory pressures and predicted percentages for pressures). In the subxiphoid group, longer duration of ECC (P= 0.028), higher pleural volume drained (P = 0.01) and higher frequency of atelectasis were observed, although these differences were not statistically significant (P = 0.10). With regard to pleural effusion (Table 3), there was a higher frequency on the left side in the subxiphoid group, both in the first (P = 0.17) and in the second (P=0.18) radiographic examination, compared with the lateral group.

**Table 2.** Perioperative data according to group [mean ± standard deviation; median (interquartile range; N [%])

	Lateral drain N = 24	Subxiphoid drain N = 24	P-value
Preoperative			
Heart rate in bpm	75 ± 10.5	72 ± 11.29	0.33^‡^
Saturation of oxygen in %	95.50 ± 1.56	96.25 ± 2.69	0.24^‡^
MIP in cmH_2_O	53.83 ± 22.71	61.46 ± 24.58	0.27^‡^
MEP in cmH_2_O	67.50 ± 25.02	73.75 ± 22.95	0.37^‡^
Percentage of predicted MIP, in %	58.93 ± 24.91	63.37 ± 20.96	0.51^‡^
Percentage of predicted MEP, in %	38.69 ± 14.13	40.86 ± 10.95	0.56^‡^
Intraoperative and IPO			
Duration of extracorporeal circulation, in minutes	113.96 ± 29.18	134.79 ± 34.32	0.028^‡^
Time in the operating room, in hours	7.06 ± 1.26	7.08 ± 1.22	0.94^‡^
Duration of mechanical ventilation, in hours	24 (20; 27)	24.5 (15; 44)	0.73*
Length of ICU stay, in hours	94.8 (71; 190)	119 (86; 181)	0.65*
Acute myocardial infarction	5 (20,8)	4 (16.7)	> 0.90**
Stroke	1 (4.2)	1 (4.2)	> 0.90**
Cardiorespiratory arrest	4 (16.6)	1 (4.2)	0.35**
Arrhythmia	12 (50)	8 (33.3)	0.38**
Postoperative			
Duration of drain use, in hours	47 (43; 55)	52.5 (45; 72)	0.11*
Drained pleural volume, in milliliters	133 (107; 171)	190 (140; 251)	0.01*
Atelectasis	1 (4.5)	6 (25)	0.10*
Respiratory infection	4 (18.2)	8 (33.3)	0.32**
Surgical wound infection	3 (13.6)	5 (20.8)	0.70**
Surgical reoperation	2 (8.3)	1 (4.2)	> 0.90**

Statistical tests used: ‡Student’s t test; *Mann-Whitney test; **Fisher’s exact test. IPO = immediate postoperative; ICU = intensive care unit; MIP = maximal inspiratory pressure; MEP = maximal expiratorypressure.

**Table 3.** Characterization of pleural effusion according to group [n (%)]

Radiographic examination 1 (48 h to 72 h after drain removal)					Radiographic examination 2 (73 h to 120 h after drain removal)				
	Absent	Small	Medium	P-value*		Absent	Small	Medium	P-value*
Right				0.64	Right				0.21
Lateral	3 (15.8)	6 (31.6)	10 (52.6)		Lateral	13 (68.4)	4 (21.1)	2 (10.5)	
Subxiphoid	3 (12.5)	11 (45.8)	10 (41.7)		Subxiphoid	12 (50)	11 (45.8)	1 (4.2)	
Left				0.17	Left				0.18
Lateral	8 (42.1)	9 (47.4)	2 (10.5)		Lateral	9 (47.7)	10 (52.6)	0	
Subxiphoid	4 (16.7)	15 (62.5)	5 (20.5)		Subxiphoid	6 (25)	16 (66.7)	2 (8.3)	

Statistical tests used: *Pearson’s chi-square test. Number of patients with lateral intercostal drain insertion who completed the study = 19. Number of patients with subxiphoid drain insertion who completed the study = 24.

Table 4 shows the differences in the predicted percentages for maximal pressures (inspiratory and expiratory) according to the time of assessment and treatment group, after adjustment for smoking, diagnoses of asthma/chronic obstructive pulmonary disease and duration of mechanical ventilation. No significant difference was observed either in inspiratory pressure (P = 0.83) or in expiratory pressure (P = 0.76), in relation to the treatment (kind of drain) at the end of the study period. In both groups, a significant reduction in the predicted percentage for inspiratory pressure was observed 24 hours after pleural drain removal (P < 0.001), and also in the predicted percentage for expiratory pressures (P <0.001). No interaction between treatment and time was detected at the end of the study period regarding the predicted values for inspiratory pressure (P = 0.09) and expiratory pressure (P = 0.17).

**Table 4.** Predicted percentages for maximal inspiratory (%PMIP) and expiratory (%PMEP) pressures according to time assessed and randomized group (mean difference ± standard error)

	Lateral	Subxiphoid	P-value*	P-value**	P-value***
%PMIP			0.83	< 0.001	0.09
24 hours - Preoperative	-34.14 ± 4.32	-32.83 ± 3.41			
72-24 hours	7.83 ± 2.10	4.42 ± 1.20			
Discharge - 72 hours	9.10 ± 1.98	6.15 ± 1.57			
%PMEP			0.76	< 0.001	0.17
24 hours - Preoperative	-18.76 ± 2.76	-20.25 ± 200			
72-24 hours	3.32 ± 1.01	2.83 ± 0.93			
Discharge - 72 hours	6.06 ± 1.34	4.29 ± 0.73			

*Difference between groups (treatment); **Difference between times; ***Treatment x time interaction. Generalized estimation equations (GEE) adjusted for smoking, diagnosis of asthma/chronic obstructive pulmonary disease and mechanical ventilation.

Both groups showed significant reductions in Borg score (thus indicating improvement in dyspnea) at the 72-hour assessment, compared with the 24-hour assessment (after adjusting for smoking and diagnoses of asthma/chronic obstructive pulmonary disease; P > 0.0001). However, no differences between the groups were seen at the end of the study period (P = 0.23). A similar result was observed in relation to the subjective degree of pain (mean Borg score), which presented a decrease (thus suggesting an improvement) at 72 hours after drain removal (group 1: 1.39; group 2: 1.89), compared with the 24-hour assessment (group 1: 2.96; group 2: 3.54), in both groups (P < 0.0001). However, there were differences between the groups at the assessment at the time of discharge (P = 0.17).

## DISCUSSION

This study evaluated the impact of insertion of pleural drains on the behavior of respiratory muscle strength, by means of a manovacuometer, in patients undergoing CABG surgery with ECC and LITA graft implantation. No significant differences in the predicted percentages for MIP and MEP at the end of the study period were observed in relation to the drain insertion site, i.e., in a lateral or subxiphoid position. On the other hand,the lateral group presented higher predicted MIP values before the time of discharge.

As expected, the patients included in this study had high prevalence of cardiovascular risk factors, along with a differentiated profile in relation to the severity of the cardiac lesions identified through coronary artery angiography. In the group of patients allocated to the lateral drain positioning, some characteristics associated with greater in-hospital cardiovascular morbidity and mortality were highlighted, and this group contained a greater proportion of individuals undergoing drug therapy. This suggests that these individuals have a profile of higher risk and vulnerability.^30^ Thus, the likelihood of occurrence of outcomes such as death and stroke in this group could be higher than among patients allocated to the subxiphoid drain.

The low predicted percentages for MIP and MEP among the patients in both groups (38 to 63%) in the preoperative assessments indicated that features associated with decreased respiratory muscle strength were present: advanced age, lung diseases and smoking; along with the progression of the cardiovascular disease itself. It is known that increased or improved maximal respiratory pressure prior to surgery is associated with fewer postoperative complications such as atelectasis, respiratory infections, duration of mechanical ventilation and length of hospital stay.^31^ On the other hand, lower maximal respiratory pressure is associated with increased incidence of cardiovascular events such as myocardial infarction, stroke and death among elderly patients.^32^

As expected and previously demonstrated,^33^ the MIP and MEP decreased significantly over the first 24 hours after the CABG and, at discharge, they still had lower values than those observed in the preoperative assessment. In previous studies in which spirometers were used as the means of assessing respiratory pressures, it was concluded that insertion of the pleural drain in the subxiphoid position was associated with lower impairment of respiratory muscle strength.^8^^,^^15^^,^^20^ However, in the present study, the pleural drain position was not found to have any significant influence on maximum respiratory pressures.

The patients allocated to group 2 had significantly longer ECC and drainage times than those in group 1. These factors are directly associated with reduced pulmonary compliance and with dysfunction,^15^^,^^34^ and this may explain our results, which were different from those of previous studies. The tendency among the patients allocated to the lateral group to present higher inspiratory pressure values before hospital discharge than those of the patients in the subxiphoid group may be partially explained by their lower BMI^35^ and by the presence of lower prevalence of atelectasis and respiratory infections and the shorter drainage time.

Unlike other studies that correlated drain insertion in the subxiphoid position with lower intensities of pain and dyspnea,^14^^,^^15^^,^^16^^,^^18^ we did not observe any differences between the groups regarding the subjectively assessed degrees of dyspnea and pain. The shorter time for which the pleural drain was used in the lateral group may have influenced these results.

Among the limitations of our study, we can cite: the sample size, which may have been insufficient after the losses; the number of deaths that occurred in only one of the groups, which may have occurred at random, but also may have represented a selection bias; and the socioeconomic (low-income) and cultural (low-education) conditions of the participants, which may also have negatively influenced our results (participants with low income and low education may have worse understanding of and compliance with medical recommendations).

## CONCLUSION

We did not observe any difference in the effect of pleural drain insertion site after CABG surgery on the respiratory pressures among patients who underwent this surgery with the use of ECC, LITA grafts and pleurotomy. A slight decline in maximal respiratory pressures in the patients with a lateral intercostal drain was identified, but with better recovery during hospitalization. In addition, higher prevalences of small and medium pleural effusion, dyspnea, respiratory infection and atelectasis in the group with a drain inserted in the subxiphoid region were observed. Despite greater initial pain sensitivity among the patients with lateral drainage, no differences were observed in relation to subjective pain and dyspnea between the groups at the end of the study. A greater number of studies conducted among candidates for CABG surgery with use of ECC and LITA grafts are needed to broaden the validation of our results.
